# Characteristics and Expression Analyses of Trehalose-6-Phosphate Synthase Family in *Prunus mume* Reveal Genes Involved in Trehalose Biosynthesis and Drought Response

**DOI:** 10.3390/biom10101358

**Published:** 2020-09-23

**Authors:** Yongjuan Yang, Kaifeng Ma, Tengxun Zhang, Lulu Li, Jia Wang, Tangren Cheng, Qixiang Zhang

**Affiliations:** 1 Beijing Advanced Innovation Center for Tree Breeding by Molecular Design, Beijing Forestry University, Beijing 100083, China; yongjuanyang_bjfu@163.com (Y.Y.); zhangtengxun@163.com (T.Z.); yuanlinlilulu@126.com (L.L.); 2 Beijing Key Laboratory of Ornamental Plants Germplasm Innovation & Molecular Breeding, Beijing Forestry University, Beijing 100083, China; makaifeng@bjfu.edu.cn (K.M.); wangjia8248@163.com (J.W.); chengtangren@163.com (T.C.); 3 National Engineering Research Center for Floriculture, Beijing Forestry University, Beijing 100083, China; 4 Beijing Laboratory of Urban and Rural Ecological Environment, Beijing Forestry University, Beijing 100083, China; 5 Engineering Research Center of Landscape Environment of Ministry of Education, Beijing 100083, China; 6 Key Laboratory of Genetics and Breeding in Forest Trees and Ornamental Plants of Ministry of Education, Beijing Forestry University, Beijing 100083, China; 7 School of Landscape Architecture, Beijing Forestry University, Beijing 100083, China

**Keywords:** trehalose biosynthesis, trehalose-6-phosphate synthase, evolution analysis, expression pattern, drought stress

## Abstract

Trehalose and its key synthase (trehalose-6-phosphate synthase, TPS) can improve the drought tolerance of plants. However, little is known about the roles of trehalose and the TPS family in *Prunus mume* response to drought. In our study, we discovered that the trehalose content in leaf, root, and stem tissues significantly increased in *P. mume* in response to drought. Therefore, the characteristics and functions of the TPS family are worth investigating in *P. mume*. We identified nine TPS family members in *P. mume*, which were divided into two sub-families and characterized by gene structure, promoter elements, protein conserved domains, and protein motifs. We found that the Hydrolase_3 domain and several motifs were highly conserved in Group II instead of Group I. The distinctions between the two groups may result from selective constraints, which we estimated by the *d_N_/d_S_* (*ω*) ratio. The ω values of all the *PmTPS* family gene pairs were evaluated as less than 1, indicating that purity selection facilitated their divergence. A phylogenetic tree was constructed using 92 TPSs from 10 Rosaceae species, which were further divided into five clusters. Based on evolutionary analyses, the five clusters of TPS family proteins mainly underwent varied purity selection. The expression patterns of *PmTPS*s under drought suggested that the TPS family played an important role in the drought tolerance of *P. mume*. Combining the expression patterns of *PmTPS*s and the trehalose content changes in leaf, stem, and root tissues under normal conditions and drought stress, we found that the *PmTPS2* and *PmTPS6* mainly function in the trehalose biosynthesis in *P. mume*. Our findings not only provide valuable information about the functions of trehalose and TPSs in the drought response of *P. mume*, but they also contribute to the future drought breeding of *P. mume*.

## 1. Introduction

Trehalose (α-d-glucopyranosyl α-d-glucopyranoside), known as a non-reducing and soluble disaccharide, has been extensively discovered in various organisms [1]. In plants, trehalose works as not only a carbon source but also as an energy signal and stress protection metabolite acting on drought, cold, heat, and freezing tolerance [2]. An impressive example is that the massive accumulation of trehalose in *Selaginella lepidophylla* and *Myrothamnus flabellifolius* (resurrection plants) allowed them to survive in an extreme desiccation environment even when 99% of water was removed from the plants [3,4]. Except for resurrection plants, trehalose has also been shown to function in the abiotic stress tolerance of rice [2], wheat [5], and tomato [6], although low trehalose content was detected. These excellent functions of trehalose in the plant kingdom mainly operate by protecting the protein and lipid membrane, as well as stabilizing the cell structure [7].

Due to the biological and physiological characteristics of trehalose, numerous studies have concentrated on the trehalose biosynthesis pathways in the response of plants to abiotic stresses at physiological, biochemical, and molecular levels. There are at least five pathways for trehalose biosynthesis [8], in which the extensively recognized pathway in plants is a two-step reaction: firstly, trehalose-6-phosphate synthase (TPS, EC: 2.4.1.15) catalyzes uridine diphosphate glucose and glucose-6-phosphate, converting to trehalose-6-phosphate (Tre6P); secondly, trehalose-6-phosphate phosphatase (TPP, EC: 3.1.3.12) prompts the Tre6P to dephosphorize into trehalose [9]. It has been found that TPS and TPP play important roles in the response of plants to environmental stimuli [10]. Further studies have reported that TPS and its product (Tre6P) are essential for plant development and improving the drought tolerance of plants by the potential function of regulating carbohydrate metabolism [11,12].

The coding genes of TPS proteins in plants have expanded into a gene family due to gene duplication events. With the completion of whole-genome sequencing, TPS family members have been collected and characterized in diverse plants; for example, 11 TPS members have been acquired from *Arabidopsis* and rice, respectively, and 12 TPSs have been obtained in *Populus* [13,14,15]. These TPS members in plants have been further divided into two sub-families: the Class I TPS sub-family, homologous to the yeast ScTPS1, and the Class II TPS sub-family, homologous to the yeast ScTPS2 [16]. Although the members of these sub-families may have originated from a common ancestor, they present distinct characteristics in terms of gene numbers, gene expression pattern, enzyme activity, and physiological function [17]. In *Arabidopsis*, TPS sub-family members (except for AtTPS3 in Class I) possess TPS1 enzymatic activity, while Class II TPS proteins are unavailable for TPS1 catalytic activity [18,19]. Inducing the heterologous or homologous expression of TPS I genes in plants may improve their tolerance for environmental stimuli; for example, the heterologous expression of *AtTPS1* in tobacco enhanced its tolerance for temperature and osmotic stresses [20]. Similarly, the transformation of rice with the *OsTPS1* increased the plant tolerance of drought, high salinity, and cold stress [21]. Beyond these roles, Class I TPS genes have been shown to be involved in strong developmental alterations and inflorescence architecture, such as stunted growth [22], aberrant root development [6], and flowering delay [12], as investigated in tobacco, tomato, and *Arabidopsis*. Based on TPS protein structure diversities of Class I and Class II, along with their function in complementing distinct TPS yeast mutants, Class II TPS members may function by distinct action modes from Class I proteins [23,24]. To explore the roles of Class II TPS members, several genes, including *AtTPS6* [25], *AtTPS5* [26], *OsTPS8* [27], and *TaTPS11* [28] have been cloned and characterized in detail. These genes can encode multifunctional enzymes with synthase and phosphatase activities, playing roles in plant architecture, stomatal closure, salt tolerance, and cold tolerance. However, the evolutionary mechanism resulting in the functional divergence between Class I TPS and Class II TPS sub-families has not yet been fully clarified. To thoroughly understand the roles and evolutionary relationships of the TPS family in plants, it is crucial to further identify and characterize TPS family genes in plants.

*Prunus mume* Sieb. et Zucc. (mei), a delightful woody plant with colorful petals, charming fragrance, and abundant flower types, has been widely domesticated in China for over three thousand years. *P. mume* is natively distributed in the Yangtze River Basin and Southwest China, although its introduction and cultivation have been further expanded in Northern China and throughout East Asia [29]. However, the water condition is one of the limiting environmental factors for the cultivation of *P. mume* further north, due to inherent climate differences. Given that trehalose and its key catalytic enzymes (TPS) play essential roles in the plant tolerance of adverse environmental conditions, research into trehalose and the TPS family is expected to be helpful for the stress tolerance breeding of *P*. *mume*. In this study, we firstly found that trehalose in the leaf, stem, and root tissues of *P. mume* significantly increased during the drought period. To explore the mechanism contributing to the increase of trehalose under drought stress, nine TPS family members were found from the genome of *P. mume*, and their gene and protein features were characterized in detail. Furthermore, analysis of the evolution of the TPS family, including selective force analysis and the identification of positively selective sites, was conducted. Finally, global expression profiles of *PmTPS* genes were examined in leaf, stem, and root tissues, as well as in response to drought stress.

## 2. Materials and Methods

### 2.1. Plant Materials and Experimental Design

*P. mume* cv. ‘Gulihong’ clones were cultivated in the plant germplasm resource center of Beijing Forestry University, Beijing, China. Two-year-old cutting seedlings were planted in plastic pots (30 cm × 28 cm × 20 cm) with peat and perlite (3:1, *v*/*v*). These seedlings were placed in a greenhouse at 25–35 °C under ambient light and 50–75% relative humidity. They were watered based on the water evaporation demand before drought stress. Then, 24 selected seedlings were divided into two groups: a normally watered control group (soil relatively water content: approximately 80%) and a drought stress group for 15 days (soil relatively water content: 29.91%) [30]. The mature leaves, tender stems, and lateral roots of the two groups were sampled in liquid nitrogen on day 0, day 3, day 7, day 12, and day 15 for subsequent trehalose measurements and qRT-PCR experiments. Roots, stems, and leaves were also acquired under normal conditions and stored at −80 °C for trehalose measurement. Each experiment was conducted with three biological replicates.

### 2.2. Relative Water Content Measurement

During the water-deficient period, soil relative water content was measured every two days in order to detect the drought degree, following the oven-drying method [31]. The relative water content of leaves was computed every two days as described by in Yang [32]. The fifth to ninth leaves from the top of a branch were harvested every two days with three biological replicates, and the fresh weight (FW) of leaves was rapidly measured. Then, the turgor weight (TW) was measured after submerging leaves in water for 8 h, and the drying weight (DW) was determined after drying at 80 °C for 24 h in an oven. Finally, the RWC of leaves was computed as (FW-DW)/(TW-DW) ×100%.

### 2.3. Trehalose Content Detection in P. mume

To monitor the changes of trehalose, the rapid detection kit for trehalose content (Nanjing Jiancheng Biology Research Institute, Nanjing, China) was applied for trehalose content measurement. Following the manufacturer’s instructions, the trehalose extracting solution was first acquired from the leaf, stem, and root tissues of *P. mume* and then used for the subsequent chromogenic reaction. After the chromogenic reaction, the BioMate 3S Spectrophotometer (Thermofisher Scientific Inc, Waltham, MA, USA) was used to detect the optical density at 620 nm. All experiments were conducted with three replicates. The collected data were finally processed using Office 2010 and the analysis of significant differences was conducted with the SPSS 22.0 (SPSS Inc., Chicago, IL, USA) software by one-way ANOVA and *t*-test.

### 2.4. Genomic Data Collection of P. mume and 11 Other Plants

The newest genome, transcripts, and protein sequences of *Arabidopsis thaliana* [33] and *Populus trichocarpa* [23] were required from Phytozome (https://phytozome.jgi.doe.gov/pz/portal.html). Data for the *P. mume* genome were derived from the *P. mume* genome website (http://prunusmumegenome.bjfu.edu.cn/) [34]. The genomic data of *P. armeniaca* [35], *P. persica* [36], *P. yedoensis* [37], *Fragaria vesca* [38], *Malus domestica* [39], *Pyrus bretschneideri* [40], *P. dulcis*, *Rosa chinensis* [41], and *Rubus occidentalis* [42] were collected from the GDR database (https://www.rosaceae.org/).

### 2.5. Identification of TPS Family Members in P. mume and 11 Other Plants

Hidden Markov models (HMM), BLASTN, and BLASTP were combined to extensively collect the TPS family sequence by searching against the genomic sequence. The Pfam seed profiles with TPS conserved protein domains (PF00982, glycosyltransferase family 20 domain, and PF02358, trehalose phosphatase domain) were derived from the Pfam database (https://pfam.xfam.org/) [43] as the source files to identify the TPS genes [44]. The HMM was built using the HMMER3.0 package and then was used for identifying the TPS proteins with two conserved domains. To further identify the TPS family members in *P. mume* and the nine Rosaceae species, BLASTP and BLASTN searches were conducted against a genome database of the 12 species by using the TPS protein and coding sequences of *A. thaliana*. Based on the BLASTN searching results, the nine genes were named *PmTPS1*, *PmTPS2*, and *PmTPS5*–*PmTPS11*.

### 2.6. Multiple Sequence Alignment and Phylogenetic Tree Construction of the TPS Family

To classify the TPS family in *P. mume*, the Cluster X 2.0.12 software (http://www.cluster-x.org/) was applied for multiple sequence alignment by using protein sequences of *P. mume*, *Arabidopsis*, and poplar; further, the MEGA 7.0 program was used to construct a phylogenetic tree with the maximum-likelihood method [45]. Additionally, 92 protein sequences of TPS from *P. mume* and 9 Rosaceae plants were aligned by Cluster X in order to explore the phylogenetic relationship of the TPS family in *P. mume* and other Rosaceae species. Then, the alignment result was used to construct a neighbor-joining phylogenetic tree using the Mega 7 software with Poisson correction methods and 1000 bootstrap replicates [46]. Meanwhile, the maximum-likelihood method was also applied to build a phylogenetic tree to validate the topologies.

### 2.7. Gene Features Analyses and Protein Subcellular Localization of TPS Family in P. mume

The TPS DNA sequences, coding sequence (CDS), gene ID number, chromosomal location, and gene length were acquired from the genomic database of *P. mume*. The TBtools software was applied to present the diagram of chromosomal location. Physical and chemical properties of TPSs, such as the amino acid number, protein molecular weight (MW), grand average of hydropathicity (GRAVY), and isoelectric point (pI), were predicted and computed online using the ExPASy tool (https://web.expasy.org/protparam/). Protein sub-cellular localization of the TPS family was conducted using the Cell-PLoc 2.0 package (http://www.csbio.sjtu.edu.cn/bioinf/plant-multi/).

### 2.8. TPS Genes Structure and Cis-Acting Elements Analyses in P. mume

The gene structures of the TPS family in *P. mume* were presented in the gene structure display server (GSDS 2.0, http://gsds.cbi.pku.edu.cn/) [47]. As predicted promoters, 2000 bp upstream sequences from ATG of *TPS*s were drawn from the genomic sequence of *P. mume*. Subsequently, the *cis*-acting elements in promoters were predicted by the Plant *Cis*-Acting Regulating Element tool (http://bioinformatics.psb.ugent.be/webtools/plantcare/html/) and diagrammed using the TBtools software.

### 2.9. Protein Motif and Conserved Domains Analysis

The MEME online tool (http://meme-suite.org/tools/meme) was applied for predicting the conserved motif structure of the TPS family in *P. mume*. To further analyze the conserved domains of PmTPSs, the protein sequences of TPS were submitted into the web-based CD-search tool of National Center for Biotechnology Information (https://www.ncbi.nlm.nih.gov/cdd). The conserved domains were visualized using the TBtools software.

### 2.10. Gene Duplication Analyses of TPS Family in P. mume

Segmental duplication genes were identified based on the Plant Genome Duplication Database (http://chibba.agtec.uga.edu/duplication/index/downloads) and sequence alignment similarity [48]. The tandem duplicated genes were determined according to Wang [49].

### 2.11. Selection Force Estimation

To estimate the selection force, synonymous (*d_S_*) and non-synonymous (*d_N_*) substitution rates, as well as the *d_N_*/*d_S_* (*ω*) ratio, of all *PmTPS*s gene pairs and each node were calculated by Phylogenetic Analysis by Maximum Likelihood (PAML) using the yn00 and codeml program after TPS sequence alignment [50]. According to the estimated values of *d_S_* and the common rate (λ) of 1.5×10^−8^ substitution per site per year for dicots, the TPS gene duplication time was computed using the following formula: T = *d_S_*/2λ × 10^6^ Mya [51]. To explore positive selection forces on amino acid sites and to analyze the molecular adaptive evolution of the TPS family, the likelihood ratio test (LRT) was used to detect the significant sites variation, which was predicted by codeml models (M3/M0 and M8/M7) in PAMLX.

### 2.12. Functional Divergence in TPS Family Protein Sequence Evolution

A previous study has reported that Type I functional divergence mainly contributed to a change of function limitation, while Type II functional divergence resulted in changes of physicochemical properties in amino acid residues [52]. To assess the functional–structural divergence of the TPS family, the DIVERGE 2.0 software was used to compute the coefficients of Type I and Type II function divergence by the maximum-likelihood method [53,54,55]. The LRT was applied for the evaluation of significance by the Chi-square test. The cutoff of Qk ≥ 0.95 was used for detecting variation of amino acid residues.

### 2.13. Expression Patterns of PmTPSs in Leaf, Stem, and Root

To explore the expression profiles of *PmTPS*s in leaf, stem, and root tissues, transcriptome data were acquired from the NCBI SRA (Genome Sequence Archive) database with accession number SRP014885. After extracting the RPKM (Reads Per Kilobase of exon model per Million mapped reads) values of every *PmTPS*s gene in three tissues, a heat map was depicted by the Heatmap Illustrator (HemI_1.0) software with default parameters.

### 2.14. Quantitative Real-Time PCR

Following the manufacturer’s instructions, a Plant RNA Extraction Kit (Omega Bio-tek, Norcross, GA, USA) was used to isolate the RNA samples from *P. mume* root, stem, and leaf tissues. After RNA purity and quality testing, the cDNA was generated using a PrimeScript^TM^ RT Reagent Kit (Takara Bio Inc., Dalian, China). The relative expression abundance of *TPS* genes was detected by setting up a 10 μL PCR reaction buffer. The PCR reaction was amplified as described by Yang [32]. The expression abundance of *PmTPS* genes was calculated by the 2^−ΔΔCT^ method, where *PmPP2A* was used as the reference [56]. Three biological replicates were performed for every experimental group. The specific primers for qRT-PCR are presented in Supplementary Materials Table S1.

## 3. Results

### 3.1. Relative Water Content Changes of Soil and Leaf Tissues during the Drought Period

**Figure 1:**
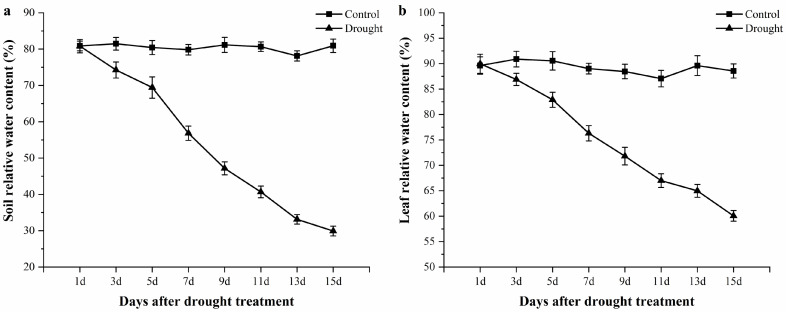
The relative water content changes of soil (**a**) and leaf tissues (**b**) during the drought period. Value is the mean of three replicates ± standard error.

For detecting the drought degree, the relative water content of soil and leaf tissues was determined once every two days. As shown in Figure 1a, the relative water content (RWC) of soil decreased from 80.87% to 29.91% over the water deficiency period. The RWC of leaves also gradually reduced, from 89.96% to 60.06%, after drought stress for 15 days (Figure 1b).

### 3.2. Trehalose Levels in Leaf, Stem, and Root of P. mume under Normal Conditions and Drought Stress

**Figure 2:**
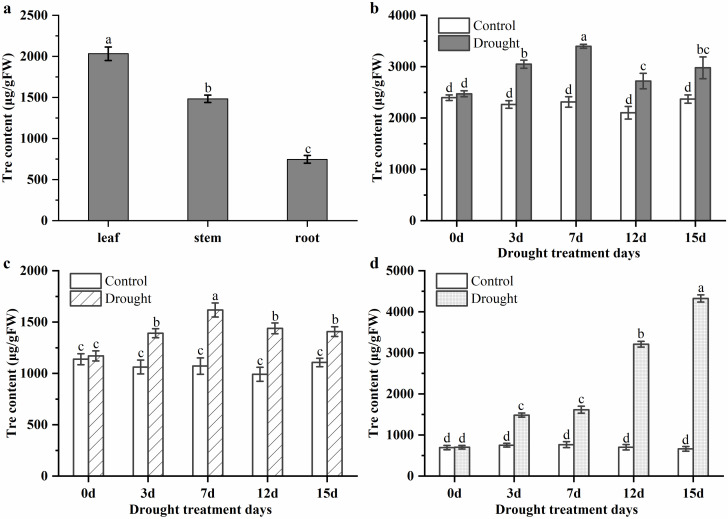
The trehalose content in the tissues (**a**) of *Prunus mume* and leaves (**b**), stems (**c**), and roots (**d**) in response to drought. Value is the mean of three replicates ± standard error. Lowercase letter represents the significant level.

To detect the trehalose level in *P. mume*, leaves, stems, and roots were selected for the determination of trehalose content. In Figure 2a, it can be seen that the trehalose levels varied in these tissues, with contents ranging from 746.67 to 2032.59 μg/gFW. Relatively high levels of trehalose were observed in leaves (2032.59 μg/gFW), followed by stems (1482.96 μg/gFW) and roots (746.67 μg/gFW).

The accumulation of trehalose could help plants enhance their tolerance for adverse environments, especially the drought, cold, and salt stress. To monitor the dynamic changes of trehalose in the response of *P. mume* to drought stress, the trehalose contents of leaves, stems, and roots were detected using a rapid detection kit for trehalose content. As shown in Figure 2b, the leaves of *P. mume* exhibited a significant accumulation of trehalose, compared with the control group, at the early stage under drought treatment. On the 7th day under drought stress, the trehalose content reached its peak: up to 1.4-fold compared with the control group. In the late stages of drought stress, the trehalose content decreased but still maintained a higher level than the well-watered group. Similar to the changes in leaves, the trehalose content of stems first increased, up to the peak on the 7th day under drought. Although the trehalose content of stems slightly decreased on the 12th and 15th days, it was still higher than that of the control group (Figure 2c). In roots, the trehalose gradually increased during the drought period, up to 6.5-fold more than that in the control group on the 15th day (Figure 2d).

### 3.3. Identification, Characteristics, and Phylogenetic Analyses of TPS Family in P. mume

**Figure 3:**
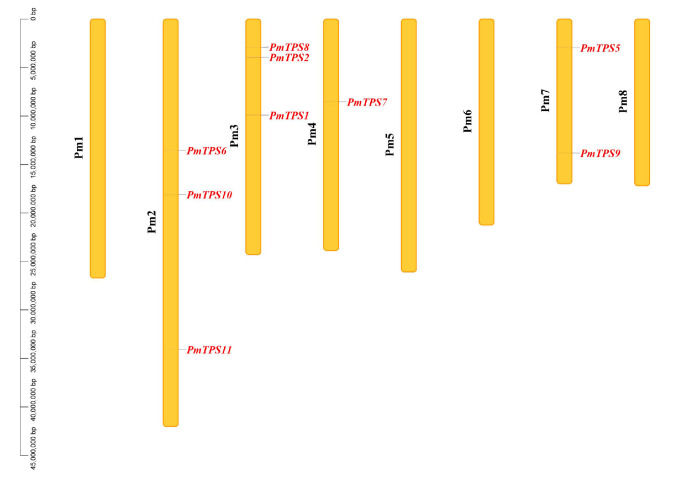
Chromosomal location of trehalose-6-phosphate synthase (TPS) family genes in *Prunus mume*. Nine TPS genes were located in four chromosomes. Pm1, Pm2, Pm3, Pm4, Pm5, Pm6, Pm7, and Pm8 represent eight chromosomes of *P. mume*, respectively.

TPS is the essential catalytic enzyme for regulating trehalose content. Therefore, nine TPS family members were identified from the *P. mume* genome by the HMMER3.0 package on the basis of Pfam seed files of PF00982 and PF02358. To further confirm the members of the TPS family in *P. mume*, BLASTN and BLASTP were performed to identify TPS family genes based on a genome-wide and protein sequence homologous search. However, this homologous search did not find new potential TPS genes for *P. mume*, aside from the initial nine TPS members. According to the genome information of *P. mume*, these nine TPS genes were distributed in the Pm2, Pm3, Pm4, and Pm7 chromosomes (Figure 3), among which *PmTPS6*, *PmTPS10*, and *PmTPS11* were located on Pm2; *PmTPS1*, *PmTPS2*, and *PmTPS8* were distributed on Pm3; *PmTPS7* was mapped on Pm4; and *PmTPS5* and *PmTPS9* were distributed on Pm7. The physicochemical properties of the nine TPS members are summarized in Table S2. Their coding protein length varied from 835 to 931 amino acids (aa), with an average length of 862.4 aa. The isoelectric points (pI) spanned from 5.67 to 6.51, and the molecular weight (MW) ranged from 94.54 to 105.26 kDa with an average molecular weight of 97.42 kDa. The prediction results of protein sub-cellular localization by the Cell-PLoc 2.0 package showed that most of the TPS proteins were located in the cytoplasm except for PmTPS1, which was located in the mitochondrion and cytoplasm.

To categorize TPS family members in *P. mume*, a phylogenetic tree was built by using MEGA 7 after the multiple sequence alignment of *A. thaliana* (11 TPS members), poplar (12 TPS members), and *P. mume* (9 TPS members); shown in Figure S1. Based on the branches of the phylogenetic tree, a total of 32 TPS members could be categorized into two sub-families: Group I and Group II. In *P. mume*, Group II was the largest sub-family, consisting of seven TPS members (PmTPS5–PmTPS11). The other two TPS members were clustered in the Group I family, which were named PmTPS1 and PmTPS2 based on their neighborhoods of *A. thaliana* TPS members.

### 3.4. Gene Structure and Promoter Cis-Acting Elements Analyses of PmTPSs

**Figure 4:**
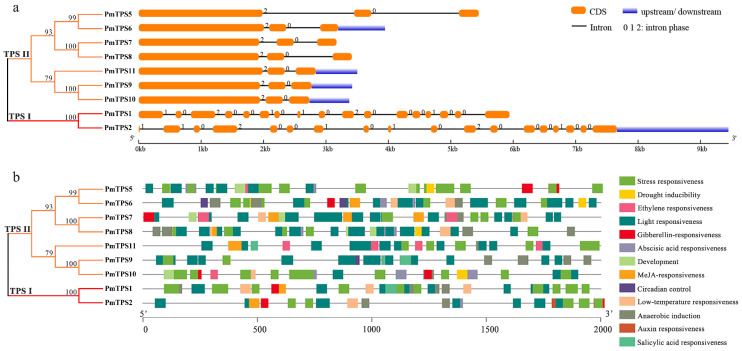
Gene structure and promoter elements of TPS genes within *P. mume*. (**a**) Exons and introns structure of TPSs in *P. mume*. The orange round-corner rectangle represents exons, the blue rectangle depicts untranslated region (UTR) regions, the black line shows introns, and numbers (0, 1, 2) represent the intron phase. (**b**) *Cis*-acting elements of the TPSs promoter in *P. mume*. Two thousand bp upstream regions of *PmTPS* genes were used for predicting *cis*-acting elements. Different color rectangles represent distinct *cis*-acting elements in response to abiotic stress, phytohormone, circadian regulation, and plant development.

To elaborate the similarity and distinction of *PmTPSs*, the intron–exon organizations of *PmTPS* genes were investigated, based on the phylogenetic tree with TPS sequences (Figure 4a). All TPS genes had more than two introns in their gene sequences, and most *PmTPS* genes harbored three exons; except for *PmTPS1* and *PmTPS2*, containing 17 and 18 exons within their gene structures, respectively. Generally, the gene structure results showed that members from the same group presented similar gene structures, especially the closest gene pairs—including the *PmTPS5*/*PmTPS6*, *PmTPS7*/*PmTPS8*, and *PmTPS9*/*PmTPS10* gene pairs—which only ranged in exon sequence length and location. Meanwhile, the intron–exon structures of members in the TPS I sub-family were significantly distinct from the TPS II sub-family. One of the TPS I sub-family members, *PmTPS2*, had the longest gene length and the most significant changes against CDS length.

The *cis*-acting elements of promoters in the gene upstream domain function by regulating the expression level of genes, especially genes in response to phytohormones and abiotic stresses. To analyze the *cis*-acting elements of *PmTPS*s promoters, 2000 bp upstream sequences from the ATG of TPS genes were extracted from the *P. mume* genome and predicted by Plant CARE. Dozens of *cis*-acting elements were identified in *PmTPS*s promoters and those in response to environmental stress and phytohormones, as well as regulating circadian rhythm and plant development, which are summarized and presented in Figure 4b. Most of these *cis*-acting elements were associated with light and stress responses. For example, G-Box, Box 4, and MRE are related to light responsiveness, while MBS, MYB, MYC, W-box, and LTR are related to responses to drought and low-temperature stresses, suggesting that *PmTPS*s positively participates in the plant response to environmental stimuli. Several *cis*-acting elements were related to phytohormones (e.g., abscisic acid, ethylene, gibberellin, auxin, kasmonic acid) responsiveness, including ABRE, ARE, ERE, GARE-motif, TGA-box, TGA-element, TCA-element, and TGACG-motif. The presence of phytohormone response elements implied that *PmTPS* genes could be induced by phytohormone signaling. Notably, *PmTPS7* and *PmTPS8* contained the most phytohormone response elements, indicating their possible function in phytohormone signal transduction.

### 3.5. Protein Domain Analyses and Multiple Sequences Alignment of TPS Family in P. mume

**Figure 5:**
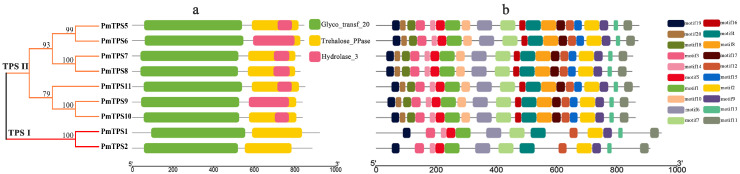
Protein conserved domains and motifs distribution of TPS family members in *P. mume*. (**a**) The protein conserved domains, glycosyltransferase 20 family (Glyco_transf_20), trehalose phosphatase (Trehalose_PPase), and haloacid dehalogenase-like hydrolase (Hydrolase_3) domains are presented by green, yellow, and pink round-corner rectangle, respectively. (**b**) Motif compositions of TPSs in *P. mume*. The distributions of 20 conserved motifs are represented by different colored boxes.

The conserved domain analysis showed that the TPS family proteins of *P. mume* consisted of two common conserved domains, including a typical glycosyltransferase 20 family (Glyco_transf_20) domain located in the N-terminal region and a trehalose phosphatase (Trehalose_PPase) domain located in the C-terminal region of the TPS protein (Figure 5a). Additionally, the haloacid dehalogenase-like hydrolase (Hydrolase_3) domain in the C-terminal region was also detected in all members of the group II TPS sub-family.

In total, 20 individual motifs of nine PmTPSs and their special distributions were presented using the MEME online program (as shown in Figure 5b). Analysis of 20 motif distributions discovered that the length of the conserved motif varied from 2 to 50 amino acid residues, and the number of PmTPSs motifs ranged from 13 to 20 in each PmTPSs protein sequence. All PmTPSs shared 13 motifs, including motifs 1–7, motif 9, motifs 11–14, and motif 19, which contained two typical conserved domains of the TPS family. The same motif permutations were observed among the TPS II sub-family members, suggesting functional similarity within the same sub-family. Compared with the TPS II family members, PmTPS1 and PmTPS2 in the TPS I sub-family lacked motif 8, motif 10, motifs 15–18, and motif 20, which might reveal that these two sub-families have experienced a divergence of gene function.

Multiple sequence alignment showed that all TPS proteins of *P. mume* presented 59.14% similarity. According to the alignment results, we found that several amino acid residues in the catalytic enzyme activity domain (Glyco_transf_20 and Trehalose_PPase domain) were highly conserved (Figure S2), indicating PmTPSs as having corresponding enzyme activities; meanwhile, the amino acid regions beyond the Glyco_transf_20 and Trehalose_PPase domains displayed relatively high diversity. These diverse regions might contribute to the functional diversity between the TPS family proteins in *P. mume*.

### 3.6. Expansion and Evolution Analyses of TPS Family Members in P. mume

Gene duplication is the main pathway to expand a gene family, which results in gene family functional divergence, including whole genome duplication, segmental duplications, and tandem duplication [57]. Based on the Plant Genome Duplication Database and our sequence alignment results, four gene pairs (*PmTPS1/PmTPS2*, *PmTPS5*/*PmTPS6*, *PmTPS7*/*PmTPS8*, and *PmTPS9*/*PmTPS10*) were identified in segmental duplications. However, no *PmTPS* genes were observed in tandem repeats. These results indicate that a segmental duplication event may have mainly contributed to the expansion of the *PmTPS* gene family.

**Figure 6:**
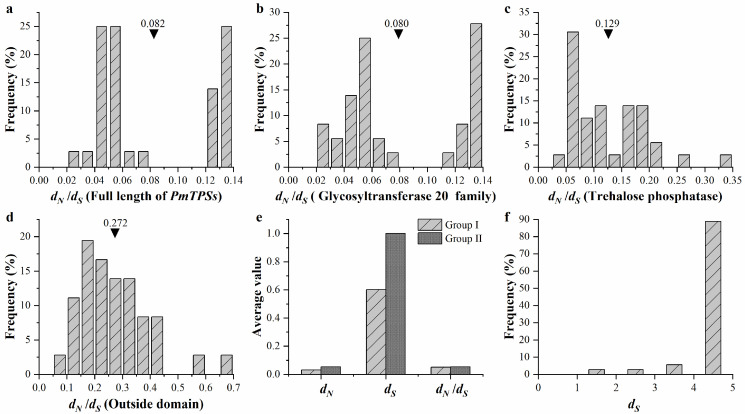
The selection pressure assessment with TPS gene pairs, genes domains, and two groups of the TPS family in *Prunus mume*. The frequency distributions of *d_N_/d_S_* for (**a**) the full length of *PmTPS*s, (**b**) glycosyltransferase 20 family, (**c**) trehalose phosphatase, and (**d**) outside domains, respectively. The black triangle represents the average value of *d_N_/d_S_* ratio. (**e**) The average value of *d_N_*, *d_S_*, and *d_N_/d_S_* in the Group I and Group II TPS family genes. (**f**) The frequency distribution of *d_S_* for TPS gene pairs in *P. mume*.

To examine the natural selection pressure in driving the gene function divergence of the TPS family in *P. mume*, the ratio of *d_N_*/*d_S_* of all gene pairs between *PmTPSs* were calculated using the yn00 program in the PAMLX software (Figure 6). The average values of all evaluated *d_N_*/*d_S_* with three domains (glycosyltransferase family 20, trehalose phosphatase, and outside domain) and the full length of the *PmTPSs* family were less than 1 (Figure 6a–d), suggesting that purity selection promoted the gene divergence of the TPS family in *P. mume*. Compared with the Glyco_transf_20 and Trehalose_PPase domains, the average value of *d_N_*/*d_S_* in the outside domain was much higher. This result indicated that the outside domain evolved faster than glycosyltransferase family 20 and trehalose phosphatase in the natural selection process. Based on the statistics of all gene pairs (Figure 6f), the distribution of *d_S_* peaked at 4.5–5, indicating that the large-scale evolution event of the TPS family in *P. mume* occurred about 150–167 million years ago. Furthermore, the estimated *d_S_* and *d_N_*/*d_S_* in Groups I and II of the TPS family in *P. mume* were determined by the codeml program using a branch model (Figure 6e). Based on the comparison of *d_S_* between Groups I and II, Cluster II of *PmTPS*s was found to have evolved earlier than Group I members.

### 3.7. Evolution and Function Divergence Analyses of Rosaceae Plants

**Figure 7:**
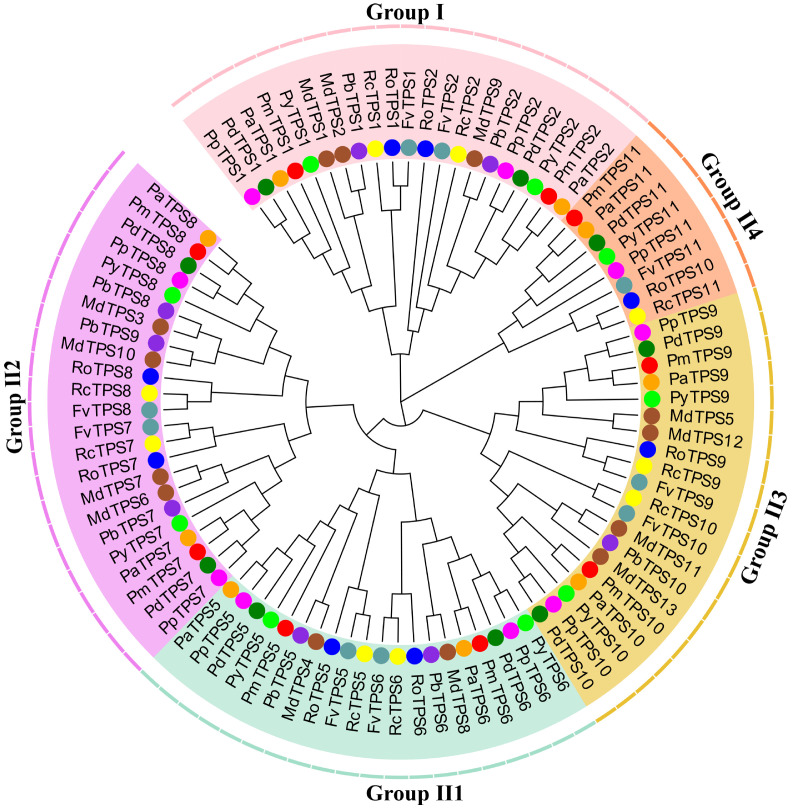
The phylogenetic tree of TPS family members from *Prunus mume* (PmTPS), *P. persica* (PpTPS), *P. yedoensis* (PyTPS), *P. armeniaca* (PaTPS), *Fragaria vesca* (FvTPS), *Malus domestica* (MdTPS), *P. dulcis* (PdTPS), *Pyrus bretschneideri* (PbTPS), *Rosa chinensis* (RcTPS), and *Rubus occidentalis* (RoTPS). The pink, #A3DFC8, violet, #E9C031, and #FF8E54 colored regions represent Groups I, II1, II2, II3, and II4, respectively. TPSs from 10 Rosaceae species are labeled by circles of red, fuchsia, lime, coral, cadet blue, sienna, green, blue violet, yellow, and blue colors, respectively. All of the referred TPS genes are listed in Table S3.

Based on the HMM and homologous searching, most Rosaceae plants contained nine TPS members, while *M. domestica* has 13 TPS members (Table S3), which may have resulted from distinct gene duplication events during the evolution process. To explore the phylogenetic characteristics of TPS family, a neighbor-joining tree was constructed using 92 TPS protein sequences from 10 Rosaceae plants. The TPS protein sequences were named and clustered into two main sub-families: Group I (21 proteins) and Group II (71 proteins). In Group I, TPSs contained two members from Rosaceae plants (except for apple, which had three Group I members). To investigate the orthologous and paralogous relationship of these TPSs, Group II sub-family members were further classified into four clusters (II1, II2, II3, and II4) with high bootstrap support, which are shown in Figure 7. This evolutionary relationship among 92 TPS members indicated that TPSs in the same cluster may have originated from a common ancestor and have similar function. It is worth noting that most PmTPSs were clustered with the TPSs of apricot, indicating that *P. mume* is more closely related to apricot.

To estimate the selective pressure acting on the TPS family division of 10 Rosacaea species, site models of positive selection were analyzed using the codeml program in PAMLX (Table 1). By likelihood ratio tests, M3 and M0 models were compared to detect the consistency of *d_N_*/*d_S_* (ω) values between codons. Under the M0 model, the estimated values of ω in five clusters were less than 1, suggesting that purity selection was the dominant selection mode acting on the TPS family. However, the chi-square test of M3 and M0 in the TPS family showed significant differences for all groups, implying that some specific sites were still altered by positive selection forces. Therefore, the comparison of models M8 and M7 was conducted to detect whether some specific codon substitutions by positive selection contributed to the TPS family gene divergences. Based on the comparison and estimation, 14, 4, 7, 13, and 16 positively selective sites were observed in the I, II1, II2, II3, and II4 groups, respectively, but only one codon site was identified under positive selection at the 95% cutoff. Our results showed no strong positive selection on amino acid sites of TPS family members among Rosaceae plants, suggesting that these TPS members were relatively conserved in the evolution process.

**Figure 8:**
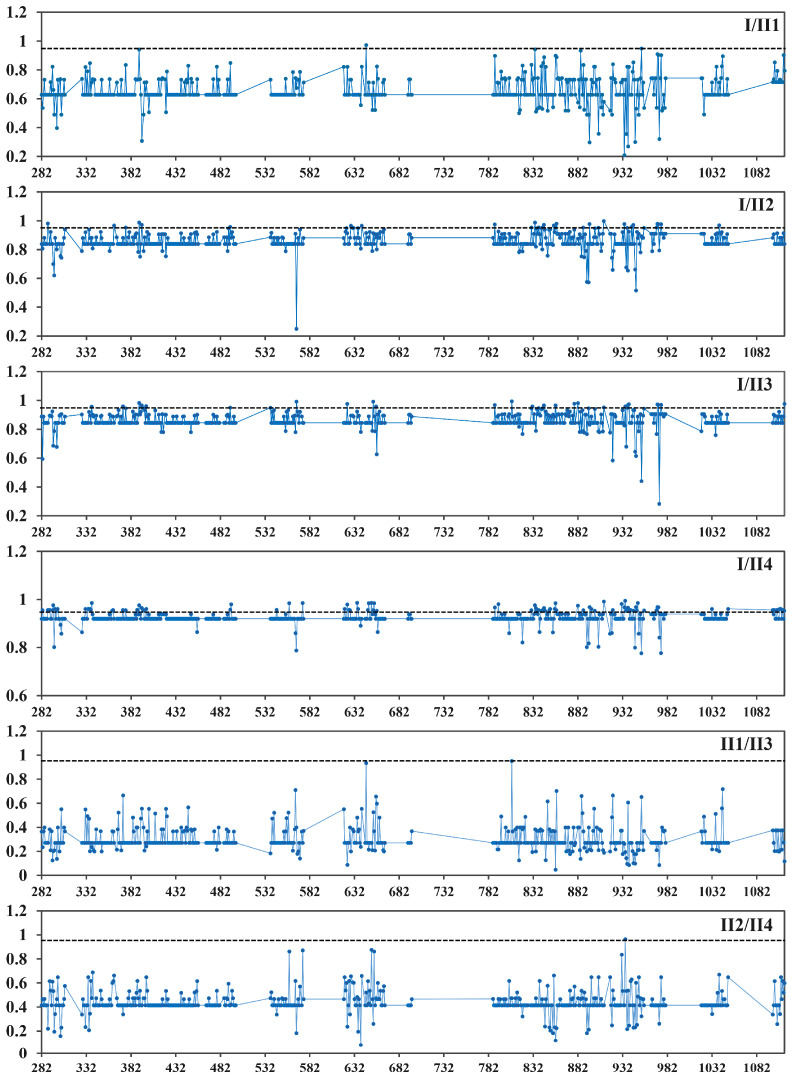
Type I functional divergence among the TPS family members of Rosacaea. Posterior probability analysis of specific amino acid sites was used for identifying critical amino acid residues. The cutoff = 0.95 is shown by the dotted line.

Type I and Type II divergence of 10 pairs from TPS I (I) and TPS II (II1, II2, II3, II4) clusters were calculated using DIVERGE 2.0 (Table S4) to exactly explore the amino acid substitutions in the TPS sub-families acting on functional divergence and the evolutionary process. On basis of the maximum-likelihood method estimation, we found that the average values of Type I coefficients (*ϴ*_I_) ranged from 0.306 to 0.927 (*p* < 0.05), among which the value of *ϴ*_I_ in I/II4 was the greatest, while that in II1/II3 was minimal. By comparison, Type II coefficients (*ϴ*_II_), which varied from 0.016 to 0.594, were less than *ϴ*_I_ of the corresponding pairwise groups. These results indicate that the Type I divergence pattern (rather than Type II) played a dominant role in the genetic functional divergence of the TPS family. We also found that the *ϴ*_I_ and *ϴ*_II_ values of these pairwise groups were less than 1, indicating that site-specific selection constraints on most TPS family genes may have led to the specific functional divergence of different groups. Therefore, posterior probability analysis of specific amino acid sites was used to identify critical amino acid residues contributing to functional divergence. Based on the posterior analysis, most amino acid sites were observed with low posterior probabilities. The cutoff value of Q_k_ ≥ 0.95 was used for detecting crucial amino acid residues resulting in Type I functional divergence between TPS clusters. Six groups with 151 sites were estimated as having posterior probabilities greater than 0.95 (Figure 8). These amino acid residues with higher posterior probabilities (Q_k_ ≥ 0.95) were considered to be devoted to the functional divergence of homologous genes. The numbers and positions of predicted amino acid substitutions varied widely among the 10 groups in the five TPS sub-families. For example, group I/II4 contained 89 critical sites, while only one member was predicted as a vital amino acid residue in groups I/II1, II1/II3, and II-2/II-4. These results indicate that functional divergence had occurred between Group I and Group II of the TPS family in 10 Rosaceae plants. Notably, most amino acid substitutions were observed in the C-terminal region (632–1113 amino acid sites) of the TPS family.

### 3.8. Expression Profile Analysis of TPS Genes in Leaf, Stem, and Root Tissues of P. mume

**Figure 9:**
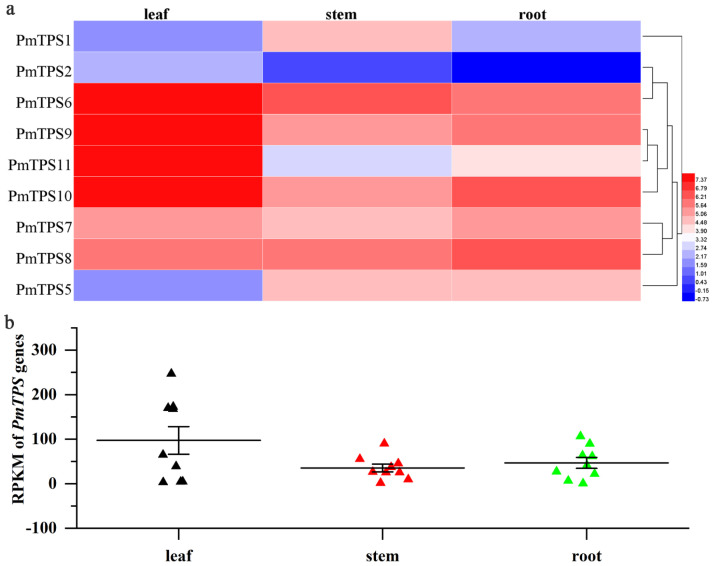
Expression patterns of *PmTPS*s among leaf, stem, and root tissues of *Prunus mume*. (**a**) The heat map represents the expression levels of nine *PmTPS*s among leaf, stem, and root tissues. Data of gene expression levels (RPKM, Reads Per Kilobase of exon model per Million mapped reads) was normalized by log_2_ transformed data. Red and blue indicate high- and low-expression profiles, respectively. (**b**) The scatter plot figure shows RPKM values of nine *PmTPS*s in leaf, stem, and root tissues.

To explore the expression pattern of *PmTPSs* in leaf, stem, and root tissues, transcriptome data of *P. mume* were acquired from NCBI. The RPKM values of *PmTPSs* were further extracted and used to draw a heat map and scatter plot figure (Figure 9a,b). In Figure 9a, we can observe the expression levels of *PmTPSs*, which exhibited divergence in three tissues of *P. mume*. A high expression level of *PmTPS6* was observed in three tissues, especially in leaf tissues. Furthermore, *PmTPS9*, *PmTPS10*, and *PmTPS11* also presented a relatively high expression in leaf tissues. It is worth noting that *PmTPS7* and *PmTPS8*, as two duplication genes, displayed a similar expression pattern in the leaf, stem, and root tissues. Distinct from other Class II TPS family members, *PmTPS5* exhibited relatively high expression level in stem and root tissues, but it had low expression level in leaf tissues. As for the members of the Class I TPS family, *PmTPS1* was highly expressed in stems, but it showed low expression in leaves and roots; meanwhile, the expression levels of *PmTPS2* in leaf tissues was higher than that in stem and root. Generally, the relative high expression levels of *PmTPS*s were observed in leaf tissues of *P. mume* (Figure 9b).

### 3.9. Expression Pattern Analysis of PmTPSs under Drought Stress

**Figure 10:**
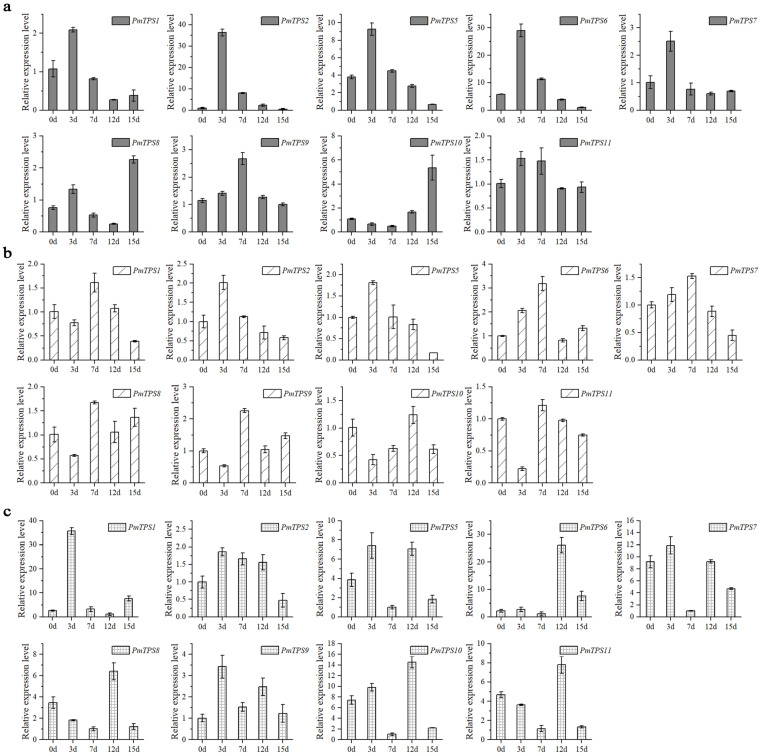
Expression patterns of TPS family genes in leaf (**a**), stem (**b**), and root (**c**) tissues of *Prunus mume* under drought stress by qRT-PCR. The abscissa axis represents water deficiency for 0d, 3d, 7d, 12d, and 15d. All qRT-PCR results are presented as mean value ± standard error from three biological replicates.

To explore the functions of the *PmTPS*s in trehalose biosynthesis and drought tolerance, the expression profiles of the nine *PmTPS*s in leaf, stem, and root tissues were detected by qRT-PCR. The expression patterns of *PmTPS*s are shown to differ in the leaf (Figure 10a), stem (Figure 10b), and root (Figure 10c) tissues of *P. mume* with the increase of drought treatment time. Although the gene expression profiles were distinct, these *PmTPS*s were upregulated and expressed to a certain degree at different drought treatment stages. In detail, *PmTPS1*, as the key enzyme of trehalose synthesis, showed significantly increased expression after drought treatment, which peaked at 3 d in leaf and root tissues. Different from its expression patterns in leaf and root tissues, the expression level of *PmTPS1* in the stem decreased at first and then increased, reaching its peak at 7 d after treatment. As for the other Class I TPS family members, *PmTPS2* in leaf, stem, and root tissues tended to positively express at first, peaking at 3 d after drought treatment. The Class II TPS family members also played crucial roles in abiotic stress tolerance by distinct function modes from Class I TPS members. The expression of *PmTPS5* in leaf and stem tissues increased in the early stage, peaked at 3 days after drought stress, and then declined; meanwhile, in root tissues, the expression of *PmTPS5* showed a substantial ‘M’ type, peaking at 3 and 7 days after treatment. *PmTPS6* and *PmTPS11* tended to show significantly increased expression at 3, 7, and 12 d after treatment in leaf, stem, and root tissues, respectively. *PmTPS7* and *PmTPS9* in leaf, stem, and root tissues were positively upregulated at 3 d and 7 d after drought treatment, while *PmTPS8* and *PmTPS10* were mainly induced at 12 d and 15 d after drought stress.

## 4. Discussion

### 4.1. Trehalose Function in P. mume Response to Drought

Trehalose, as an osmoprotectant, has been proven to rapidly accumulate under cold, salt, and drought stress, in order to protect plants from damage. A typical example is the ability of high levels of trehalose in *M. flabellifolius* and *S. lepidophylla* (3% and 12% of dry weight, respectively) to rescue plants during extreme dehydration stress [3,58]. However, the trehalose content in other plants has been detected in relatively low levels. For example, 10 μg/gFW trehalose was determined in *Arabidopsis* [12]; while 17 and 15 μg/gFW trehalose contents were detected in transgenic rice [2] and tobacco [59], respectively, by overexpressing the *TPS* gene. In *P. mume*, 746.67-2032.59 μg/gFW trehalose was measured in leaves, stems, and roots, which were much higher than that in *Arabidopsis*, transgenic rice, or transgenic tobacco. This suggests the function of trehalose in the drought tolerance of *P. mume*, which is consistent with the results of a similar study on cassava [60].

Although the trehalose level was relatively low, an increase of trehalose in plants could enhance their abiotic stress tolerance. For example, the trehalose accumulation improved drought tolerance in rice [2], the trehalose content of cassava was up to 2.3–5.5 fold during an osmotic stress period [60], and the higher levels of trehalose in *desi* chickpea genotypes led to better drought stress tolerance [61]. In our study, the trehalose contents in leaf, stem, and root tissues of *P. mume* significantly increased during drought stress, indicating that the trehalose was responsible for the response of *P. mume* against drought stress.

### 4.2. Characteristics of TPS Family in P. mume

TPS, known as the crucial catalytic enzyme in trehalose accumulation, plays a vital role in abiotic stress tolerance and plant development. The genome-wide identification and characterization of TPS family genes have been conducted in *Arabidopsis* [14], rice [15], poplar [13], potato [62], lotus [63], apple [64], cotton [65], and *Moringa oleifera* [44]. There have been no systematic studies focused on the biological functions of the TPS family in *P. mume*. By searching the *P. mume* genome, nine TPS family members were identified and characterized in *P. mume*. Previous studies have suggested that the numbers of TPS family members vary in plants; for example, 11 TPS genes have been identified in *Arabidopsis* [14], 12 TPS members have been obtained in poplar [13], and 13 TPS genes have been acquired from apple [64]. Compared with *Arabidopsis*, rice, poplar, and apple, our results indicated a decrease of TPS members in *P. mume*, which may have been caused by ancient polyploidy events. Multiple amino acid sequence alignment showed that the sites in the glycosyltransferase 20 family and trehalose phosphatase domains of TPS members in *P. mume* were highly conserved, suggesting that the structures of the two domains were basically founded before genetic functional divergence. Our results resembled the characteristics in *Arabidopsis*, rice, poplar, and drumstick tree [13,44]. Based on the multiple sequence alignment and phylogenetic analysis, the nine PmTPSs were further clustered into two groups, similar to the division of AtTPSs in *Arabidopsis* [14]. Several distinct characteristics of the two groups were observed, in terms of conserved motifs and gene structures. Motif 8, motif 10, motifs 15–18, motif 20, and the Hydrolase_3 domain were relatively conserved in Group II TPS genes, compared to Group I; meanwhile, the number of exons in Group I was remarkably greater than that in Group II TPS genes. These characteristic distinctions suggest that the two groups of genes underwent different selective constraints and functional divergence. Compared with Group I, the average values of *d_S_* and *d_N_*/*d_S_* in Group II genes were higher but less than 1, suggesting that Group II genes evolved faster than those of Group I under more relaxed purity selection. These evolution modes between Group I and Group II have also been observed in drumstick tree [44]. A recent document has reported that Class I TPS sub-family members, except for *AtTPS3*, possess TPS1 enzymatic activity (unlike Class II TPS genes) in *Arabidopsis* [19]. In our study, *PmTPS1* and *PmTPS2* were identified as belonging to the group I TPS family and showed high similarity with *AtTPS1* in terms of conserved domains. The results suggest that the functions of *PmTPS1* and *PmTPS2* might resemble those of *AtTPS1*; however, the exact functions of *PmTPS1* and *PmTPS2* need to be further explored in *P. mume*.

### 4.3. Functional Evolution Analyses of TPS Family in Rosaceae

In this study, 92 *TPS* family genes were identified in *P. mume*, *P. persica*, *P. yedoensis*, *P. armeniaca*, *F. vesca*, *M. domestica*, *P. dulcis*, pear, *R. chinensis*, and *R. occidentalis*. The TPS family members of these species ranged from 8 to 13, indicating they have experienced different gene duplication events. For example, recent whole-genome duplication occurred in apple 50 million years ago [39], while apricot and *P. mume* did not undergo genome-wide duplication events; however, large segmental duplications have occurred in their genomes [34,35]. Based on the phylogenetic analysis, the 92 genes from 10 Rosaceae species were divided into two main sub-families (Group I and Group II), which is consistent with the classification in *Arabidopsis*, rice, and poplar [13]. To explore the homologous relationships, Group II sub-families were further classified into four clusters—Groups II1, II2, II3, and II4—similarly to the classification in winter wheat [66] and drumstick tree [44]. These genes in the same cluster have originated from a common ancestor and share similar functions. However, what caused the division of the TPS family needs further exploration and explanation. To explore the mechanisms of TPS family gene functional divergence, branch mode was used to detect the selection forces among five groups, which showed that the estimated values of *ω* in the five clusters were varied and less than 1. Meanwhile, site mode analyses revealed that the significant positive selection constraint had acted on specific codon sites in the gene evolution process. These results imply that the ranged purity selection would lead to different evolution rates, dividing the TPS family genes into five groups, while positive selection mainly functioned on the fixation of amino acid mutations to enable gene function diversity after gene duplication. This is consistent with studies in sunflower and soybean, which have reported that positive selection forces could drive the divergence of gene function in the same clades by accelerating the retention of favorable amino acid mutations [67,68]. Evolutionary analyses in soybean [68], potato [62], lotus [63], poplar, and salix [69] have revealed that amino acid substitutions can result in genetic functional divergence [53,70]. Therefore, the genetic functional divergence of the TPS family was analyzed by DIVERGE to identify the crucial amino acid substitutions. The coefficient analysis of divergence types showed that Type I divergence played a major role in gene function divergence of these plants, resulting from some key amino acid substitutions. By posterior probability analysis, 151 amino acid residues in the 10 group pairs were identified as the key sites for most TPS family gene diversification, leading to group-specific functional divergence. Most of the amino acid sites were located in the C-terminal region of TPS proteins. Amino acid substitutions in the C-terminal domain also hint at genetic functional divergence.

### 4.4. Roles of PmTPSs in Trehalose Biosynthesis in P. mume

During the drought period, plant systems positively maintain physiological water balance by increasing water absorption in root tissues, decreasing the water loss in stem and leaf tissues, and adjusting biosynthesis process of osmotic protectant in tissues [71]. Trehalose, as an osmotic protectant, has been widely explored in the root, stem, and leaf tissues of plants [2]. In our study, we found that the level of trehalose was higher in leaf tissues, followed by stem and root tissues. Meanwhile, *PmTPS2*, *PmTPS6*, *PmTPS9*, and *PmTPS11* exhibited high correlation with the trehalose level in leaf, stem, and root tissues, suggesting that these genes might participate in regulating the biosynthesis of trehalose in *P. mume* under normal conditions.

At the beginning of the study, we found that trehalose could rapidly accumulate in the leaf, stem, and root tissues of *P. mume* under drought stress. TPSs have been reported as the key enzyme in the biosynthesis of trehalose [17]; however, only the Class I sub-family has TPS catalytic activity [19]. Recently, some members of TPS II have been reported to affect the trehalose content; for example, the *PvTPS9*-RNAi transgenic root nodules of bean altered the expression level of TPS II members and resulted in a significant decrease of trehalose in root nodules [72]. In this study, the expression of *PmTPS2*, *PmTPS5*, *PmTPS6*, *PmTPS9*, and *PmTPS11* in leaf tissues was induced on day 3 and day 7 after drought stress, as correlated with the increase of the trehalose content in leaf tissues under drought stress. This result indicates that *PmTPS2*, *PmTPS5*, *PmTPS6*, *PmTPS9*, and *PmTPS11* may be the key enzymes for trehalose biosynthesis in the leaf tissues of *P. mume*. The expression level of *PmTPS2*, *PmTPS6*, and *PmTPS7* in stem increased on day 3 and day 7 after water deficiency, which was related to the changes of trehalose in stem tissues under drought. In root tissues, *PmTPS2 and PmTPS6* were upregulated on day 12, which was consistent with the increase of trehalose level in root tissues. Generally, the expression levels of *PmTPS2* and *PmTPS6* were highly correlated with the changes of trehalose content in leaf, stem, and root tissues, showing that *PmTPS*2 (Group I) and *PmTPS6* (Group II) in *P. mume* may play roles in drought tolerance by participating in trehalose biosynthesis, as consistent with the analysis between trehalose content and the expression level of *PmTPSs* under normal condition. Overall, the accumulation of trehalose and the positive expression of *PmTPSs* help *P. mume* tolerate drought stress.

## 5. Conclusions

In summary, a high level of trehalose was detected in distinct tissues of *P. mume*, where the accumulation of trehalose in *P. mume* could help it withstand drought stress, indicating that trehalose is essential for the drought tolerance of *P. mume*. TPS is the critical enzyme for trehalose biosynthesis. Therefore, we identified nine TPS family members in *P. mume* and provided a comprehensive analysis of their gene structure, protein conserved domains, and selective constraints. A phylogenetic tree of 92 TPS members from *P. mume* and nine other Rosaceae species was constructed, from which the 92 TPS members could be classified into five groups. The expression patterns of *PmTPS*s under drought indicated that the TPS family helped *P. mume* against drought. Combined with the changes of trehalose and expression profiles of *PmTPSs* under normal conditions and drought, we found that the TPSs in *P. mume* may play their roles in drought tolerance by participating in trehalose biosynthesis. Given the importance of trehalose and the TPS family in the response of *P. mume* to drought, our study provides valuable insights into the functions of trehalose and *PmTPSs* in the drought tolerance of *P. mume* and lays a foundation for the drought breeding of *P. mume*.

## Figures and Tables

**Table 1:** Detection selective pressure of the TPS family under site models.

Group	N ^a^	*d_N_/d_S_*(ω) under M0 ^b^	2Δ/M3vs.M0 ^c^	2Δ/M8vs.M7	M8 Estimates ^d^	Selective Position ^e^
I	21	0.11642	333.308 **	4.551342	p1 = 0.00414 ω = 2.40953 (p = 0.22333 q = 1.52122)	5V,13Y,32K,37L,424S,437A,545K*,549K,673T,674D,675T,700P,701V,707N
II1	20	0.06074	184.367 **	0.01504	p1 = 0.00001 w = 42.36549 (p = 0.28727 q = 3.69465)	68S,302S,317M,321R
II2	11	0.09378	282.973 **	0.045086	p1 = 0.00376 w = 1 (p = 0.27992 q = 2.27746)	3L,54S,305I,314Q,411L,435S,497G
II3	20	0.12158	276.959 **	0.01508	p1 = 0.00001 w = 16.38964 (p = 0.28482 q = 1.78922)	10A,29S,30T,240Q,251S,332P,373L,378L,380I,382E,455A,709S,712S
II4	8	0.15098	92.617 **	6.04069	p1 = 0.00808 w = 3.5522 (p = 0.29967 q = 1.6545)	42S,43H,67S,84K,109S,311Q,357F,387Q,455D,456R,651A,785S,790V,834L,837V,843G

^a^ The number of sequences in the group. ^b^ *d_N_*/*d_S_*(*ω*) under M0 represents the ratio under the branch model. ^c^ Significance test is conducted by chi-square test, in which double asterisks (**) represents *p* < 0.01 and one asterisk (*) represents *p* < 0.05. ^d^ ω is computed under the M8 model, p1 is the predicted proportion of positively selective sites, p and q are the parameters of the beta distribution. ^e^ The positively selective sites in the amino acids are listed under M8 by Bayes Empirical Bayes (BEB) analysis.

## Supplementary Materials

The following are available online at https://www.mdpi.com/2218-273X/10/10/1358/s1, Table S1: Specific primers of TPS family members and reference gene in *Prunus mume*, Table S2: Information and characteristics of TPS family members in *Prunus mume*, Table S3: The TPS genes used to reconstruct phylogenetic trees, Table S4: Function divergence between subgroups of TPS family in Rosaceae plants, Figure S1: Phylogenetic tree of 32 TPS protein sequences from *Arabidopsis*, poplar and *Prunus mume*, Figure S2: Multiple sequences alignment of 9 TPS family members in *Prunus mume*.
