# Supplementary material for: Characteristics and Expression Analyses of Trehalose-6-Phosphate Synthase Family in Prunus mume Reveal Genes Involved in Trehalose Biosynthesis and Drought Response
Source: Biomolecules. 2020 Sep 23;10(10):1358. doi: 10.3390/biom10101358 (PMC7598203; doi:10.3390/biom10101358)
Supplement: Supplementary file 1 [file biomolecules-10-01358-s001.pdf]

Supplementary Material

Characteristics and Expression Analyses of Trehalose-6-Phosphate Synthase Family in *Prunus mume* Reveal Genes Involved in Trehalose Biosynthesis and Drought Response

Yongjuan Yang [1,2,3,4,5,6,7], Kaifeng Ma [2,3,4,5,6,7], Tengxun Zhang [1,2,3,4,5,6,7], Lulu Li [1,2,3,4,5,6,7], Jia Wang [2,3,4,5,6,7], Tangren Cheng [2,3,4,5,6,7] and Qixiang Zhang [1,2,3,4,5,6,7,*]

1 Beijing Advanced Innovation Center for Tree Breeding by Molecular Design, Beijing Forestry University, Beijing 100083, China; yongjuanyang_bjfu@163.com (Y.Y.); zhangtengxun@163.com (T.Z.); yuanlinlilulu@126.com (L.L.)
2 Beijing Key Laboratory of Ornamental Plants Germplasm Innovation & Molecular Breeding, Beijing Forestry University, Beijing 100083, China; makaifeng@bjfu.edu.cn (K.M.); wangjia8248@163.com (J.W.); chengtangren@163.com (T.C.)
3 National Engineering Research Center for Floriculture, Beijing Forestry University, Beijing 100083, China
4 Beijing Laboratory of Urban and Rural Ecological Environment, Beijing Forestry University, Beijing 100083, China
5 Engineering Research Center of Landscape Environment of Ministry of Education, Beijing 100083, China
6 Key Laboratory of Genetics and Breeding in Forest Trees and Ornamental Plants of Ministry of Education, Beijing Forestry University, Beijing 100083, China
7 School of Landscape Architecture, Beijing Forestry University, Beijing 100083, China
* Correspondence: zqxbjfu@126.com; Tel.: +86-01062338005

1 SUPPLEMENTARY DATA

Table S1 Specific primers of TPS family members and reference gene in *Prunus mume*
Table S2 Information and characteristics of TPS family members in *Prunus mume*
Table S3 The TPS genes used to reconstruct phylogenetic trees
Table S4 Function divergence between subgroups of TPS family in Rosaceae plants
Figure S1 Phylogenetic tree of 32 TPS protein sequences from *Arabidopsis*, poplar and *Prunus mume*
Figure S2 Multiple sequences alignment of 9 TPS family members in *Prunus mume*

2 SUPPLEMENTARY TABLES AND FIGURES

2.1 SUPPLEMENTARY TABLES

Table S1. Specific primers of TPS family members and reference gene in *Prunus mume.*

Gene ID	Forward primers	Reverse primers
PmTPS1	TGAGAGAAAGGGAGCTAAGG	CTGTAAGTCAGGCTCAAATGG
PmTPS2	CTGCTGATTTGGTTGGTTTCC	GAGCTTTCCTTGATCCTCTACTC
PmTPS5	GGCACGAGAAGCATTATAGG	CAATCACCCTGAATCCTAACC
PmTPS6	GGAATGCGAAATTGGATAAGG	TTGCTCCACTCAAAGAAGG

PmTPS7	TGGTCTACCTGAAGAGATGG	GGAACACACTTGAACCTATCC
PmTPS8	TGTAGCAAACCAACTTCCC	TAGCAAAGCATCCTCATTCC
PmTPS9	GTTAATGCTGTGAGGGATGG	GGGAATCAGAGGCAAATCC
PmTPS10	CATCTGTTTGTCGTGAGAGG	ACTGAAGCGCCATTTATCC
PmTPS11	GAACCCAGATGAGGATTTCG	GAAGAAGGAAAGGGACTATGC
PmPP2A	ATATAGCTGCTCAGTTCAACC	AAAAACAGTCACCACATTCTT

Table S2. Information and characteristics of TPS family members in *Prunus mume*

Gene name	Gene ID	Gene Length (bp)	ORF Length (bp)	Protein Length (aa)	Exons	Introns	PI	MW (kDa)	GRAVY	Predicted Subcellular localization
PmTPS1	Pm011257	5940	2796	931	17	16	6.39	105.26	-0.411	Cytoplasm; Mitochondrion
PmTPS2	Pm010337	7660	2685	894	18	17	6.51	100.34	-0.243	Cytoplasm
PmTPS5	Pm023183	5448	2577	858	3	2	5.67	97.35	-0.184	Cytoplasm
PmTPS6	Pm005885	3194	2568	855	3	2	5.84	96.92	-0.194	Cytoplasm
PmTPS7	Pm014011	3169	2517	838	3	2	5.81	95.70	-0.29	Cytoplasm
PmTPS8	Pm010161	3414	2508	835	3	2	5.85	94.54	-0.274	Cytoplasm
PmTPS9	Pm024703	2767	2541	846	3	2	6.41	96.31	-0.189	Cytoplasm
PmTPS10	Pm006639	2732	2541	846	3	2	5.77	95.58	-0.225	Cytoplasm
PmTPS11	Pm008557	2835	2580	859	3	2	6.37	97.71	-0.206	Cytoplasm

Table S3. The TPS genes used to reconstruct phylogenetic trees

Organism	Sequence	Gene identifier	Database
Arabidopsis thaliana	AtTPS1	At1g78580	Phytozome
	AtTPS2	At1g16980	
	AtTPS3	At1g17000	
	AtTPS4	At4g27550	
	AtTPS5	At4g17770	
	AtTPS6	At1g68020	
	AtTPS7	At1g06410	
	AtTPS8	At1g70290	
	AtTPS9	At1g23870	
	AtTPS10	At1g60140	
	AtTPS11	At2g18700	
Populus trichocarpa	PtTPS1	estExt_fgenesh4_pg.C_1680018	Phytozome
	PtTPS2	e_gw1.IV.2524.1	
	PtTPS3	fgenesh4_pg.C_LG_III000738	
	PtTPS4	grail3.0010065002	
	PtTPS5	estExt_Genewise1_v1.C_290287	
	PtTPS6	estExt_Genewise1_v1.C_LG_X6311	
	PtTPS7	eugene3.00110684	
	PtTPS8	fgenesh4_pg.C_LG_IV000367	
	PtTPS9	fgenesh4_pm.C_LG_XII000278	
	PtTPS10	eugene3.00150531	
	PtTPS11	eugene3.00061363	

	PtTPS12	fgenesh4_pm.C_LG_XVIII000320	
Prunus mume	PmTPS1	Pm011257	*P. mume* genome project
	PmTPS2	Pm010337	
	PmTPS5	Pm023183	
	PmTPS6	Pm005885	
	PmTPS7	Pm014011	
	PmTPS8	Pm010161	
	PmTPS9	Pm024703	
	PmTPS10	Pm006639	
	PmTPS11	Pm008557	
Prunus persica	PpTPS1	Prupe.4G155900.1	Genome Database for Rosaceae
	PpTPS2	Prupe.4G071400.1	
	PpTPS5	Prupe.5G031500.1	
	PpTPS6	Prupe.1G334900.1	
	PpTPS7	Prupe.3G113100.1	
	PpTPS8	Prupe.4G067300.1	
	PpTPS9	Prupe.5G176400.1	
	PpTPS10	Prupe.1G256200.1	
	PpTPS11	Prupe.1G095500.1	
Prunus armeniaca	PaTPS1	PARG05688m02	Genome Database for Rosaceae
	PaTPS2	PARG11982m01	
	PaTPS5	PARG23082m01	
	PaTPS6	PARG06888m02	
	PaTPS7	PARG15117m03	
	PaTPS8	PARG12017m01	
	PaTPS9	PARG24673m02	
	PaTPS10	PARG06151m02	
	PaTPS11	PARG24333m02	
Prunus dulcis	PdTPS1	Prudul26A004570P1	Genome Database for
	PdTPS2	Prudul26A019681P1	
	PdTPS5	Prudul26A011250P1	
	PdTPS6	Prudul26A018374P1	
	PdTPS7	Prudul26A001342P2	
	PdTPS8	Prudul26A030173P1	
	PdTPS9	Prudul26A010062P2	
	PdTPS10	Prudul26A026998P1	
	PdTPS11	Prudul26A017236P2	
Prunus yedoensis	PyTPS1	CYE_r3.1SPE4_g012720.1	Genome Database for
	PyTPS2	CYE_r3.1SPA4_g007710.1	
	PyTPS5	CYE_r3.1SPA5_g006960.1	
	PyTPS6	CYE_r3.1SPE1_g026410.1	
	PyTPS7	CYE_r3.1SPA3_g013030.1	
	PyTPS8	CYE_r3.1SPA4_g007340.1	
	PyTPS9	CYE_r3.1SPE5_g023590.1	
	PyTPS10	CYE_r3.1SPE1_g017950.1	
	PyTPS11	CYE_r3.1SPE0_g002670.1	
Pyrus bretschneide	PbTPS1	XM_009378449.2	Genome Database for
	PbTPS2	XM_009362524.2	
	PbTPS5	XM_009372922.2	

	PbTPS6	XM_009353290.2	
	PbTPS7	XM_009367004.2	
	PbTPS8	XM_009353349.2	
	PbTPS9	XM_018646691.1	
	PbTPS10	XM_009365245.2	
Fragaria vesca	FvTPS1	FvH4_3g17650.1	Genome Database for
	FvTPS2	FvH4_3g06590.1	
	FvTPS5	FvH4_5g22550.1	
	FvTPS6	FvH4_4g33380.1	
	FvTPS7	FvH4_6g27760.1	
	FvTPS8	FvH4_3g07260.1	
	FvTPS9	FvH4_5g05100.1	
	FvTPS10	FvH4_4g34800.1	
	FvTPS11	FvH4_4g09780.1	
Rosa chinensis	RcTPS1	XM_024302706.1	Genome Database for
	RcTPS2	XM_024342775.1	
	RcTPS5	XM_024315251.1	
	RcTPS6	XM_024338945.1	
	RcTPS7	XM_024322648.1	
	RcTPS8	XM_024303657.1	
	RcTPS9	XM_024319215.1	
	RcTPS10	XM_024337423.1	
	RcTPS11	XM_024337311.1	
Rubus occidentalis	RoTPS1	Ro03_G18602	Genome Database for
	RoTPS2	Ro03_G05334	
	RoTPS5	Ro05_G30925	
	RoTPS6	Ro04_G00245	
	RoTPS7	Ro06_G24984	
	RoTPS8	Ro03_G33090	
	RoTPS9	Ro05_G12139	
	RoTPS10	Ro04_G27454	
Malus domestica	MdTPS1	MDP0000139096	Genome Database for
	MdTPS2	MDP0000267875	
	MdTPS3	MDP0000278633	
	MdTPS4	MDP0000838058	
	MdTPS5	MDP0000135837	
	MdTPS6	MDP0000919905	
	MdTPS7	MDP0000856196	
	MdTPS8	MDP0000913703	
	MdTPS9	MDP0000227381	
	MdTPS10	MDP0000265728	
	MdTPS11	MDP0000257194	
	MdTPS12	MDP0000799020	
	MdTPS13	MDP0000258781	

Table S4. Function divergence between subgroups of TPS family in Rosaceae plants

	Type I				Type II	
**Pairs**	**θ_I±SE[a]**	**LRT**	***P*-value**	**Q_k≥0.95[b]**	**θ_{II}±SE[c]**	***P*-value**
I/II1	0.6616±0.093	51.120455	0.0000	1	0.522935±0.036	0
I/II2	0.8608±0.082	110.717588	0.0000	33	0.594027±0.034	0
I/II3	0.863113±0.087	99.321567	0.0000	26	0.56001±0.034	0
I/II4	0.926695±0.109	72.862902	0.0000	89	0.575447±0.031	0
II1/II2	0.4104±0.079	27.235426	0.0000	0	0.080003±0.044	0.0692028
II1/II3	0.3056±0.071	18.382175	0.0000	1	0.016402±0.042	0.7028359
II1/II4	0.452±0.108	17.364483	0.0000	0	0.089717±0.039	0.0202918
II2/II3	0.4536±0.072	39.682501	0.0000	0	0.101465±0.043	0.0187773
II2/II4	0.4424±0.094	22.187962	0.0000	1	0.109542±0.040	0.0060384
II3/II4	0.46087±0.109	17.72313	0.0000	0	0.076643±0.039	0.0468983

[a] The coefficient value of Type I function divergence with its error.

[b] The number of amino acid sites under posterior probability ($Q_k \geq 0.95$).

[c] The coefficient value of Type II function divergence with its error.

2.2 SUPPLEMENTARY FIGURES

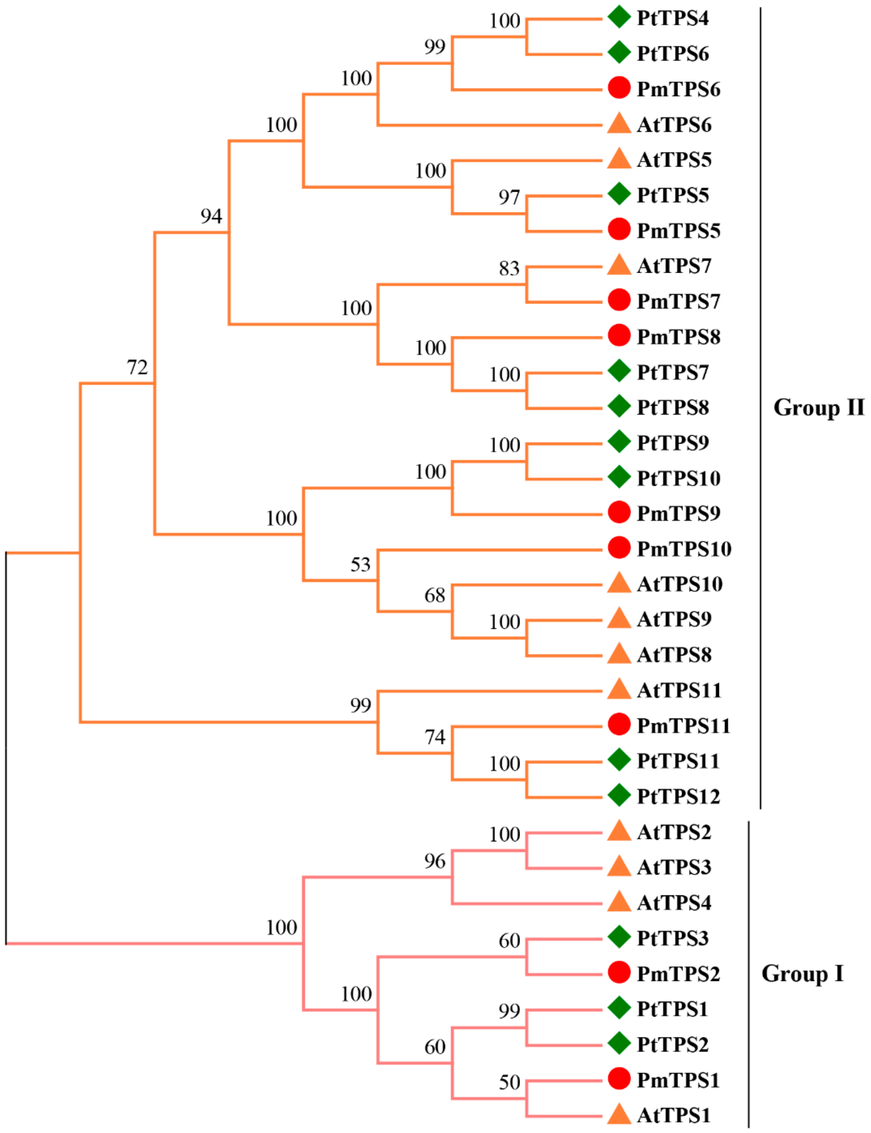

Figure S1. Phylogenetic tree of 32 TPS protein sequences from *Arabidopsis*, poplar and *Prunus mume*. The red branch represents the group I subfamily of TPSs and the orange branch depicts the group II subfamily of TPSs. The red dots represent the TPSs of *P. mume*, the orange triangles show the TPSs in *Arabidopsis* and the green diamonds depict the TPSs of poplar.

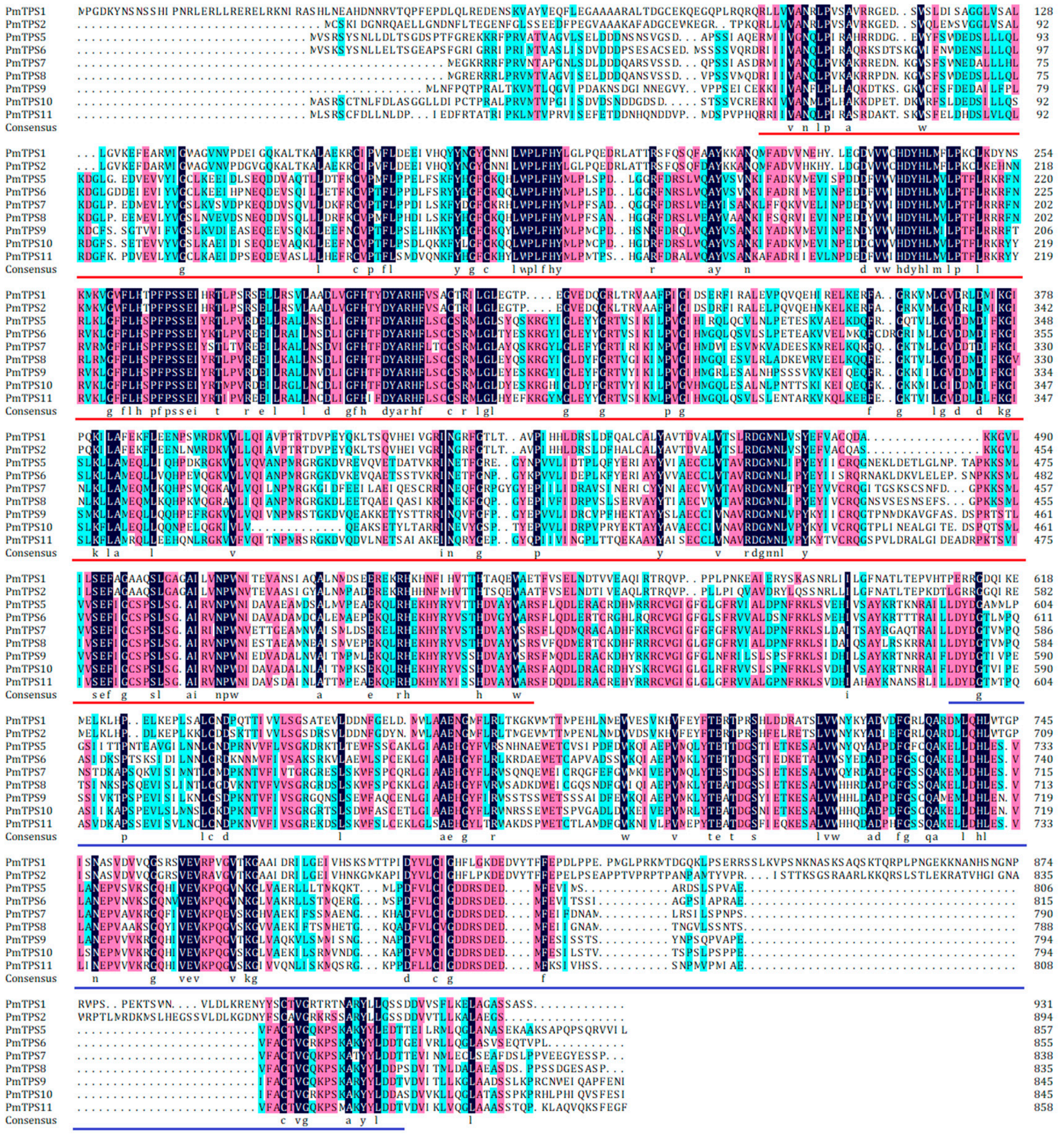

Figure S2. Multiple sequences alignment of 9 TPS family members in *Prunus mume*. The red line represents the glycosyltransferase family 20 domain and the blue line depicts the trehalose phosphatase domain.
